# Physicochemical Profile of Canastra Cheese Inoculated with Starter Cultures of *Kluyveromyces lactis* and *Torulaspora delbrueckii*

**DOI:** 10.3390/foods14010121

**Published:** 2025-01-03

**Authors:** Adriele do Amor Divino Silva, Dérica Gonçalves Tavares, Rafaela Pereira Andrade, Tamara Leite dos Santos, Whasley Ferreira Duarte

**Affiliations:** 1 Departament of Biology, University of Lavras (UFLA)—Campus Universitário, Lavras 37200-900, MG, Brazil; adriele2704@gmail.com (A.d.A.D.S.); rafaelaandrade1210@gmail.com (R.P.A.); tamaraleitesantos@gmail.com (T.L.d.S.); 2 National Institute of Coffee Science and Technology, Departament of Biology, University of Lavras (UFLA)—Campus Universitário, Lavras 37200-900, MG, Brazil; derica.goncalvestavares@louisville.edu

**Keywords:** probiotic yeasts, functional foods, artisanal cheese

## Abstract

Canastra cheese, an artisanal cheese produced in Serra da Canastra—Brazil, has great cultural importance. Furthermore, this cheese has nutritional and sensory attributes that make it of great economic importance. Its microbiota is composed of different bacteria and yeasts. Some yeasts already isolated by our research group have been characterized as potential probiotics. Probiotic microorganisms have garnered scientific interest, as improvements in the physical, chemical and sensory characteristics of food products have been reported when these microorganisms are used. In this context, the objective of this work was to evaluate *Kluyveromyces lactis* and *Torulaspora delbrueckii*, which were previously isolated from Canastra cheese, as autochthonous starter cultures. Canastra cheese was produced under three different conditions: (1) cheese with “Pingo” (natural starter), (2) cheese with “Pingo” + yeast mixed culture, and (3) cheese with only mixed yeast culture. The results showed that the mixed yeast inoculum significantly influenced the lactic acid bacteria population. Yeast populations remained at around 106 CFU/g after 45 days of maturation. Furthermore, cheeses containing the yeast mixed with inoculum had an initial lactose content reduced by 92.80% compared to cheese produced with “Pingo” (87.70%). The antioxidant activity, evaluated using the ABTS method, showed that cheeses containing the mixed yeast culture had higher percentages of antioxidant activity at 45 days of maturation. The texture profile of the cheeses changed over time. In general, the cheese containing the yeast mixed culture and “Pingo” and the cheese containing “Pingo” had the lowest hardness at 30 days of maturation (5245 and 5404 N, respectively). Among the volatile compounds, 3-methylbutyl octanoate, phenethyl butyrate, phenethyl propionate, isobutyl butanoate and pentyl propionate were found only in cheeses produced with yeast mixed culture. The obtained results demonstrated that the use of autochthones probiotic cultures could improve the cheese characteristics without negatively impacting the traditional physicochemical attributes of Canasta cheese.

## 1. Introduction

Minas artisanal Canastra cheese is a type of artisanal cheese produced in the Serra da Canastra region in the state of Minas Gerais, Southwest Brazil. This cheese is produced from raw milk using a culture of endogenous microorganisms called “Pingo”. “Pingo” is obtained from the cheese whey collected after the second salting of the cheese produced the previous day and contains lactic bacteria, such as those of the *Lactobacillus* and *Enterococcus* and *Weissela* genera, and yeasts, such as *Candida*, *Debaryomyces*, *Pichia*, *Kluyveromyces*, *Torulaspora*, *Yarrowia*, *Wickerhamiella*, *Trichosporon*, among others. Due to the diversity of microorganisms capable of fermenting milk, some small producers use Pingo as a starter in the production of Canastra cheese [1,2,3].

Canastra cheese has great cultural and economic importance and is the main source of income for small producers in the region [3]. It is estimated that approximately 800 families depend on the sale of this product. In 2019, the profit obtained from the sale of Canastra cheese was approximately US $20 million. This demand was met only due to intense production equivalent to 20 kilos/day/producer in the previous year [2].

Compared to other Brazilian artisanal cheeses, Canastra cheese requires a longer maturation period, and according to legislation, Canastra cheese must mature for at least 22 days at room temperature before being sold. This maturation period, in addition to promoting greater safety in consumption, contributes to greater microbial diversity [2,4].

During the maturation period, the cheese’s water activity decreases, and the cheese’s endogenous microorganisms produce some organic acids that are responsible for the low pH and increased acidity. Together, these factors create an unfavorable environment for colonization by pathogenic microorganisms [2]. These characteristics, which are linked to the climate, temperature, altitude, native pasture and solar incidence of the region, give this cheese unique sensory characteristics [1,5].

Lactic acid bacteria play an important role during the cheese maturation process, contributing to lactose fermentation and lactic acid production, in addition to being involved in the biochemical processes that result in desirable sensory characteristics. However, more recent studies have reported the importance of other endogenous microorganisms, such as yeast.

Yeasts are common microorganisms in cheese production ecosystems due to their tolerance to a wide range of pH, temperature, low water activity and high salt concentrations. Yeasts can metabolize lactose and galactose and assimilate lactic and citric acids, which is important in the reestablishment of Gram-positive bacteria that are sensitive to acidic environments [6,7]. They play an important role in the microbiota and ripening of cheese, as they consume lactic acid with a consequent increase in pH. This factor favors the emergence of proteolytic bacteria [8,9]. In general, the most common yeasts found in the most diverse types of cheese are *Kluyveromyces marxianus*, *Debaryomyces hansenii*, *Saccharomyces cerevisiae*, *Yarrowia lipolytica* and *Rhodotorula mucilaginosa*.

Although some yeasts are associated with undesirable characteristics, such as fruit flavor, mucoid texture and changes in color, in cheese, others improve the texture and aroma profile through the production of important metabolites [9]. In Brazil, yeasts have been the target of some studies in recent years due to the need for a better understanding of their role in the quality of cheese. Andrade et al. [1] isolated and identified *Torulaspora delbrueckii* B14 and *Kluyveromyces lactis* B10, and subsequent works by our group have evaluated their impact as simple and mixed inocula in cheese production. Andrade et al. [10] evaluated the impact of *T. delbrueckii* B14 and *K. lactis* B10 as simple and mixed inocula for cheese production, and also the survival capacity under simulated gastrointestinal conditions. These authors observed that yeasts presented a survival rate greater than 80% after passage through the simulated gastrointestinal tract, had self-aggregation rates greater than 90% and exhibited β-galactosidase activities of 0.35 U/g and 0.53 U/g, respectively. Both yeasts survived the maximum concentration of 10% NaCl for 21 days and showed growth at 4 °C. Andrade et al. [11] demonstrated the ability of the yeast *K. lactis* B10 to maintain the survival of mice up to 24 days after infection with salmonellosis, with a survival rate of 90%. This survival rate, in comparison with the protection exerted by *Saccharomyces boulardii* (70%), demonstrate that the use of these yeasts in food products for probiotic purposes is very promising.

The few studies on yeast in cheese have shown that these microorganisms play an important role in the cheese maturation process. Furthermore, the use of potentially probiotic yeasts as starter cultures in the production cheese is understudied and may provide several promising applications. Thus, the objective of this research was to evaluate the effects of using the yeasts *K. lactis* B10 and *T. delbrueckii* B14 as autochthonous starter cultures and their impact on the physicochemical characteristics of Canastra cheese.

## 2. Materials and Methods

### 2.1. Preparation of the Inoculum

*K. lactis* B10 and *T. delbrueckii* B14 were previously isolated from Canastra cheese and stored at −80 °C in 40% glycerol. The reactivation of the yeasts was carried out in YPD (1% yeast extract; 2% peptone; 2% dextrose) and incubation at 28 °C for 24 h. The inoculum was multiplied until its population reached a cell concentration of 8 Log cells/mL. After obtaining the desired population, the yeasts were centrifuged at 10,000× *g* at 4 °C for 7 min and stored in peptone water (0.1% *w*/*v*) until use.

### 2.2. Cheese Production

The cheeses were produced in a cheese factory in the municipality of Bambuí—MG (20°0′21″ S, 45°58′ 37″ W), using the producer’s physical infrastructure, milk and methods with the aim of faithfully reproducing the Canastra cheese production process. The raw milk was collected immediately after milking, transported to the cheese factory and then processed. A total of 150 L of raw milk, at approximately 35 °C, was transferred to manufacturing tanks and then the specific inoculum for each treatment was added. Three treatments, in triplicate, were evaluated: I—Cheese made only with the producer’s starter “Pingo”; II—Cheese made with “Pingo” and the mixed inoculum *K. lactis* B10 and *T. delbrueckii* B14; III—Cheese made using only the yeast mixed inoculum.

Milk coagulation was performed with commercial rennet (HA-LA^®^ Chr. Hansen Lavras, Brazil) at a concentration of 1 mL/L (75 IMCU/mL). After adding the inoculum, rennet and salt to each treatment, the inoculum was mixed with the milk for 5 min followed by rest for 30 min. The obtained curd was cut with a stainless-steel cutter and homogenized for 5 min. Then the whey was removed, and the cheese mass was added to the molds. After molding, the cheeses were salted via dry salting, where the salt was deposited on the cheese surface. After 24 h, the cheeses were removed from the molds and stored in maturation chambers at room temperature (18 to 22 °C), turning every day to ensure product uniformity. The cheeses were matured over 45 days and samples were collected every 15 days. The protein was 29.61–30.12%, the moisture was 38.54–40.12% and the fat was 31.0–31.8%.

### 2.3. Monitoring the Yeast and Bacteria Population During Cheese Maturation

Yeast and bacterial populations were monitored in the cheese during the cheese maturation process at 0, 15, 30 and 45 days. For plating, 5 g samples of cheese were diluted in 45 mL of 0.1% sterile peptone water and homogenized by vortexing. Subsequent dilutions were then prepared by transferring 100 μL of the previous solution to tubes containing 900 μL of 0.1% peptone water in labeled tubes for subsequent plating.

For yeast counting, 100 μL aliquots were plated on plates containing YPD medium with 0.01% (*w*/*v*) chloramphenicol and incubated at 28 °C for 48 h. To count bacteria, plates containing MRS medium supplemented with 0.2% (*v*/*v*) nystatin were used and incubated at 37 °C for the same period. After incubation, the population was determined, and the results were expressed in forming units per gram of cheese (Log CFU/g).

### 2.4. Determination of Sugars and Acids by HPLC

Acids and sugars were analyzed by liquid chromatography and determined in g/kg. To extract sugars, 0.5 g of cheese was diluted in 2.5 mL of mobile phase and vortexed. After extraction, the material was centrifuged at 10,000 rpm for 10 min at 4 °C and the supernatant obtained was filtered through 0.22 µm filters. The chromatograph used was the Shimadzu (Shimadzu Corp, Kyoto, Japan) equipped with a refractive index detector (RID-10A). The column used was Supelcogel 8H (Supelco, Bellefonte, PA, USA) (7.8 mm × 30 cm) at 30 °C. The mobile phase used was a 0.005 M H_2_SO_4_ solution at a flow rate of 0.5 mL/min. Identification was carried out by comparing the retention times of the compounds with those of pure standards injected under the same conditions. The concentrations of the identified compounds were determined by external calibration according to Andrade et al. [1]. All analyses were performed in triplicate.

### 2.5. Determination of Total Phenolic Compounds

Total phenolic compounds were evaluated by the Folin–Ciocalteu method according to Kamtekar et al. [12]. For this purpose, extracts were prepared according to the methods of Gil et al. [13], with modifications. Briefly, 0.5 g of cheese was diluted in 40 mL of methanol, resulting in an extract with a concentration of 10 mg/mL. This extract remained in the ultrasonic bath for 10 min and was then vortexed for one minute. Subsequently, 0.75 mL of the extract was added to 1.25 mL of 10% (*v*/*v*) Folin–Ciocalteu solution and 1 mL of 4% (*w*/*v*) sodium carbonate solution. The mixture was homogenized in a vortex mixer and left to rest for 2 h in the dark. The samples were analyzed spectrophotometrically at 750 nm. The total phenolic content was expressed as mg of gallic acid equivalent per g of extract (EAG/g).

### 2.6. Antioxidant Activity of Cheeses—ABTS Test

The DPPH and ABTS•+ radical scavenging methods were carried out according to the methods described by Gil et al. [13]. The samples were kept in a dark environment, and spectrophotometric analyses were subsequently carried out. The results are expressed as percentage of free radical scavenging.

### 2.7. Electron Microscopy

The cheese samples collected at 15, 30 and 45 days of maturation were fixed in modified Karnovsky (composed of 2.5% glutaraldehyde, 2.5% formaldehyde in 0.05 M sodium cacodylate buffer (pH 7.2, 0.001 M CaCl_2_) and kept in the refrigerator for a week) for a minimum of 24 h. Then, the samples were washed three times for 10 min each with sodium cacodylate buffer (0.05 M) and dehydrated in an increasing acetone gradient (25%, 50%, 75%, 90%, once and 100% three times) for approximately 10 min in each solution. After this dehydration, the samples were placed in the Balzers CPD 030 critical point device to replace acetone with CO_2_ and complete drying. After this process, the samples were mounted on aluminum stubs with double-sided carbon tape on an aluminum foil film and covered with gold in an evaporator (Sputter Coater SCD 050—Balzers). The electron micrographs were recorded digitally under working conditions of 20 kV and a working distance of 9 mm using a Tescan Clara field emission scanning electron microscope from the Electronic Microscopy and Ultrastructural Analysis Laboratory (LME, UFLA).

### 2.8. Texture Profile Analysis (TPA)

TPA of the cheeses was carried out on a TA-XT2i SMS texturometer (Stable Micro Systems Ltd., Surrey, UK) equipped with a 75 mm diameter cylindrical probe. The hardness (N), adhesiveness (N), cohesiveness (N) and gumminess (N) of the samples were analyzed. The following parameters were used: cheese sample volume, 2 cm^3^; compression time, 5 s; test speed, 5 mm/s; and posttest speed, 2 mm/s [14]. Six measurements were taken for each sample.

### 2.9. Analysis of Volatile Organic Compounds by HS–SMPE GC–MS

The volatile compounds from the cheese samples were extracted using the solid phase microextraction technique (HS SPME), as described by Menezes et al. [15], with modifications. Samples (3 g) were placed in 20 mL vials. A 50/30 μm divinylbenzene/carboxene/polydimethylsiloxane (DVB/CAR/PDMS) fiber supplied by Supelco (Bellefonte, PA, USA) was used to extract the volatile compounds. The fiber was stabilized for 10 min at 65 °C and then exposed in vials containing the sample for 25 min at the same temperature. After extraction, the fiber was kept for 5 min in the injector at 230 °C to desorb volatiles.

Volatile compounds were analyzed by gas chromatography-mass spectrometry (GC-MS) (Model GCMS-QP2010; Shimadzu, Tokyo, Japan) equipped with a Carbowax column (30 m × 0.25 mm id. × 0.25 μm). The oven temperature was set at 40 °C for 5 min, increased to 220 °C (at a rate of 10 °C/min) and finally held at this temperature for 10 min. The carrier gas was high-purity helium at 0.7 mL/min. The mass detector operated in selective mode was a quadrupole with an electron impact ionization system at 70 eV and 250 °C. Volatile compounds were identified using the NIST 2011 library and identities were confirmed using the linear retention index (LRI) calculated from the injection of a series of alkanes (C8–C40). The relative percentage of compounds was calculated by normalizing the peak area.

### 2.10. Statistical Analysis

The obtained data were subjected to ANOVA and Tukey’s test. A heatmap was used to visualize the volatile organic compound data. All analyses were carried out using R software version 4.3.3.

## 3. Results and Discussion

### 3.1. Microbial Population Changes Throughout the Canastra Cheese Maturation Process

**Figure 1:**
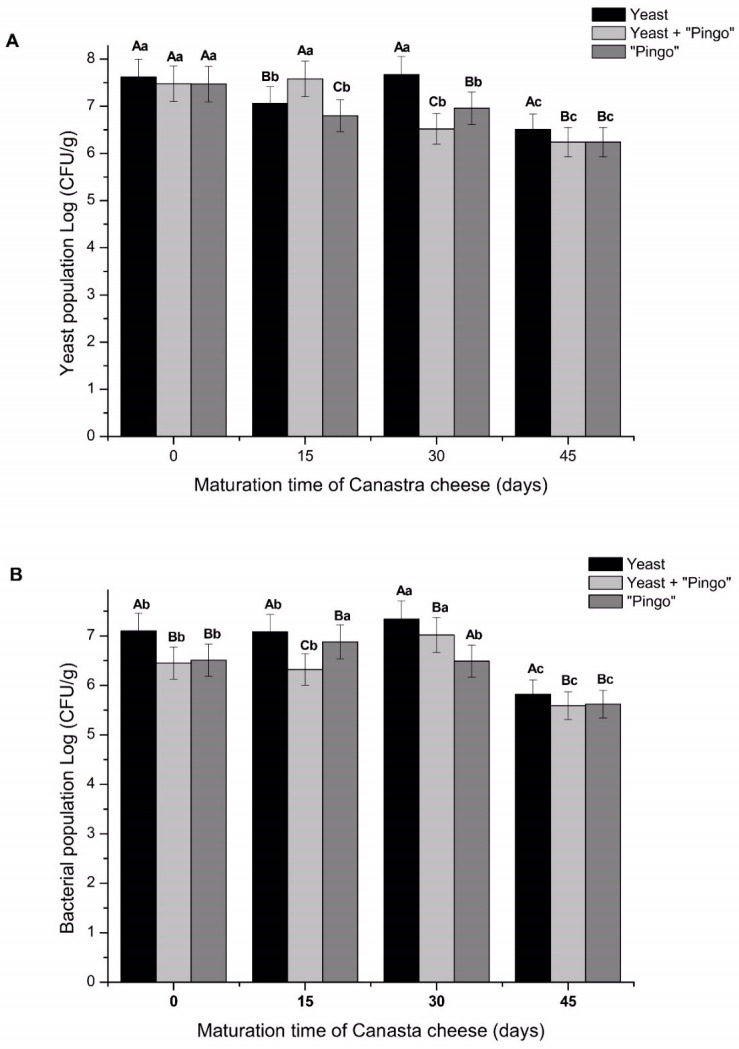
Microbial population over 45 days of cheese maturation. (**A**)—Yeast population. (**B**)—Bacterial population. The data are expressed as the mean of triplicate samples ± standard deviation. Means with different letters are significantly different according to the Tukey test, *p* < 0.05. Capital letters represent the breakdown of treatments within time, and lowercase letters represent the breakdown of times within the treatment.

The yeast population in the cheeses was monitored for 45 days of maturation (Figure 1). At 15 days of maturation, the yeast + “Pingo” cheese had the highest yeast count (7.58 log CFU/g). However, at 30 days of maturation, there was a significant increase (*p* < 0.05) in the yeast population for the yeast mixed inoculum cheese. In this case, the yeast population was greater (7.67 log CFU/g) in relation to “Pingo” cheese (6.96 log CFU/g) and yeast + “Pingo” cheese (6.52 log CFU/g) (Figure 1A).

At 45 days of maturation, the yeast mixed inoculum cheese maintained the highest yeast population (6.51 log CFU/g) compared with the yeast + “Pingo” and “Pingo” cheeses, which did not differ from each other (6.24 and 6.25 log CFU/g, respectively). The yeast population in Canastra cheese has been previously reported in the literature by Andrade et al. [1] and Kothe et al. [16]. In the latter, the authors found that the diversity and abundance of yeast present in cheeses produced on several farms in the Serra da Canastra region were relatively high. Considering the yeast population present in “Pingo” and that in the used yeasts mixed culture, a higher yeast count in yeast + “Pingo” cheese could be expected. However, throughout the maturation of cheese, competition between yeasts and bacteria for nutrients can influence the reduction of the yeast population [17]. This hypothesis can be confirmed with the largest yeast populations being observed in cheeses containing separate inocula (yeast mixed inoculum or “Pingo” cheese).

Regarding the bacterial population (Figure 1B), it was observed that the highest bacterial population count was found for the yeast mixed inoculum cheeses (7.08 log CFU/g) followed by “Pingo” (6.88 Log UFC/mL) and yeast + “Pingo” (6.32 Log UFC/g). At 30 days, the yeast mixed inoculum and the yeast + “Pingo” cheese were those with the highest populations (7.34 and 7.02 Log CFU/g, respectively). Finally, at 45 days of maturation, the yeast mixed inoculum cheese presented the highest population (5.82 Log CFU/g), while the yeast + “Pingo” and “Pingo” cheeses did not show a significant difference between populations (5.59 and 5.62 Log CFU/g).

The results suggest that inoculation of *K. lactis* B10 and *T. delbrueckii* B14 in the cheese did not inhibit the growth or survival of lactic acid bacteria, since the population of bacteria in yeast mixed inoculum cheese was significantly greater (*p* < 0.05) at all analyzed maturation times than in cheeses with no added mixed yeast inoculum (Figure 1B). Similar results were observed for the yeast + “Pingo” cheese. This same synergistic effect was observed by Centeno et al. [17], who associated the presence of *K. lactis* with an increase in *Enterococcus faecium* counts throughout the maturation of Cebreiro-type cheese. This phenomenon may be due to the release of growth factors such as amino acids and vitamins through autolysis or excretion, which promote the growth of bacteria. Furthermore, the release of some organic compounds by yeast, as well as the use of lactic acid during yeast metabolism, can result in an increase in pH, favoring the emergence of the bacterial community [18,19].

Another factor observed was the reduction in yeast and bacteria populations in all cheeses after 45 days of maturation (Figure 1A,B). Some factors related to the characteristics of cheese can limit the viability of microorganisms. Some of these factors are low water activity due to the loss of water in the cheeses during maturation, an increase in salt concentration and a significant change in pH. Together, these factors create, over time, an inhospitable environment for some microorganisms, resulting in a general tendency toward a decrease in microbial populations with an increase in the maturation period [2].

Considering that the yeasts evaluated in this work were previously characterized as potentially probiotic, it is worth mentioning that to exert probiotic effects, a food must contain between 8 and 9 log/CFU, which corresponds to the intake of approximately 100 g of food that contains between 6 and 7 log/CFU [20]. Our results are in line with expectations, since at the end of 45 days of maturation, all cheeses had yeast populations above 6 log CFU/g product. These results suggest good stability of the yeast population, even when used in combination with other microorganisms present in “Pingo”, allowing their use without significantly negative changes to the product in question.

### 3.2. Determination of Sugars and Acids by HPLC

Organic sugars and acids contribute to the development of the flavor and aroma of a wide variety of cheeses [21]. Sugar analysis of the cheeses revealed that the lactose content decreased in the first 15 days in the yeast mixed inoculum cheese, which was equivalent to 92.88% of the initial lactose content, while the lactose content in the “Pingo” cheese decreased to 87.70% of the initial content. The yeast + “Pingo” cheese, on the other hand, showed a reduction in lactose content to 92.80% of the initial content. At 30 days of maturation, the lactose content of the yeast mixed inoculum cheese decreased to approximately 50% of the lactose content measured at 15 days. On the other hand, yeast + “Pingo” and “Pingo” cheese did not show variations in relation to the previous time (15 days). At 45 days of maturation, the lactose concentration remained approximately constant in all cheeses compared to the concentrations found at 30 days of maturation (Table 1). The yeast mixed inoculum cheese presented the lowest residual lactose concentration at 45 days; however, the differences observed were nonsignificant (*p* > 0.05). These results, taken together, show that the introduction of mixed yeast culture significantly improves lactose consumption from cheese directly, since *K. lactis* yeast is already reported as a strain capable of metabolizing lactose, and indirectly through stimulation of BAL growth [19]. However, it is known that the interaction between bacteria and yeast during cheese ripening is a complex process and natural competition between these microorganisms may have been responsible for the lower consumption of lactose in cheeses containing both inoculums.

Carbohydrates can be used by microorganisms as precursors for their metabolism and, consequently, for their multiplication, which is in agreement with data referring to the yeast population (Figure 1A), where, notably, there is an increase in population concomitant with a reduction in the lactose content at 30 days of maturation (Table 1). Some previous work has demonstrated the efficiency of *K. lactis* strains in metabolizing lactose compared to other yeast strains or even lactic acid bacteria [1].

Glucose and galactose were not detected immediately after cheese production or during maturation. Generally, glucose and galactose do not appear in cheese analyses at the beginning of production. The residual concentrations of these sugars found after a few days of maturation are generally related to the degradation of lactose. In this case, the emergence and consumption of glucose and galactose likely occurs between sampling intervals [10].

Organic acids are important in the physicochemical characteristics of the cheese matrix, preventing the appearance of pathogenic microorganisms and improving sensory aspects [22]. In milk, these acids are present naturally and originate from bovine biochemical metabolism, lipolysis or microbial metabolism, which may explain the concentration of lactic acid at the zero time of cheese maturation [23].

Among the acids analyzed, lactic acid is the main acid responsible for the low pH of cheese and directly influences parameters such as microbial population, texture and flavor [22,24]. Yeast mixed inoculum cheese showed an increase in lactic acid in the first 15 days of maturation concomitant with a decrease in lactose. However, at subsequent maturation times (30 and 45 days), the concentration of this acid progressively decreased (Table 1). On the other hand, the lactic acid concentrations observed in the yeast + “Pingo” and “Pingo” cheeses were greater than those in the yeast mixed inoculum cheese at 30 and 45 days. This factor may have been caused by the greater population of lactic acid bacteria in cheeses that contained “Pingo” as an inoculum. Similar results were found by Garde et al. [21], who suggested in their work that the presence of homofermentative bacteria can cause high concentrations of lactic acid during cheese maturation.

The acetic acid concentrations in the cheeses increased up to 15 days of maturation, with a subsequent decrease in all the treatments after maturation (Table 1). Citric acid concentrations decreased between 0 and 45 days in all cheeses produced (Table 1). These results suggest that citric acid metabolism may be responsible for the emergence of acetic acid [22].

### 3.3. Analysis of Total Phenolic Compounds and Antioxidant Activity Measured by the ABTS Method

**Figure 2:**
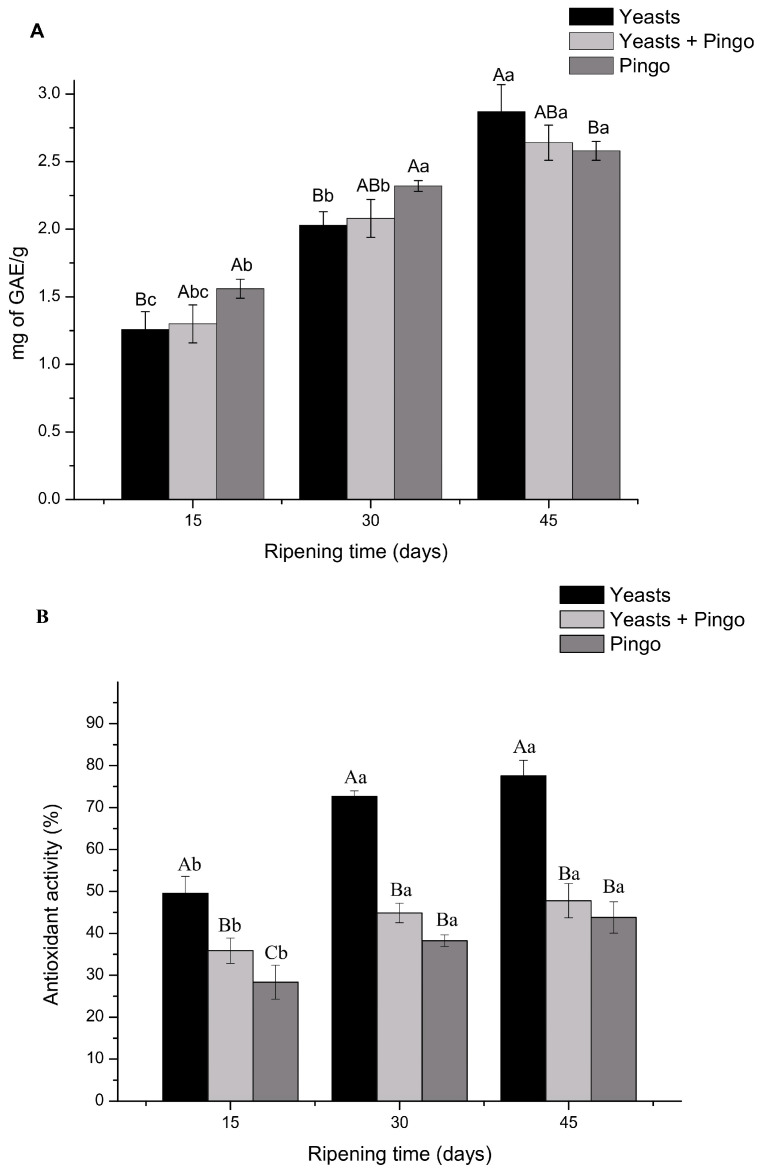
Phenolic concentration and antioxidant activity were evaluated using the Folin–Ciocalteu and ABTS methods for yeast mixed inoculum cheeses, “Pingo” and mixed inoculum and “Pingo after 15, 30 and 45 days of maturation. Data are expressed as the mean of triplicates ± standard deviation. Means with different letters are significantly different according to Tukey’s test, *p* < 0.005. Capital letters indicate distribution of treatments over time. Lowercase letters indicate times within the treatment. (**A**) TFC—Total phenolic compounds (mg GAE/g). (**B**) Positive control for ABTS.

Given the importance of polyphenols in the human body, the phenolic compounds in cheeses at different maturation times were evaluated. According to the results, all cheeses showed a significant increase (*p* < 0.05) in the concentration of phenolic compounds over time. However, at 15 and 30 days of maturation, “Pingo” cheese presented higher concentrations of phenolic compounds (1.56 and 2.32 mg GAE/g, respectively) than yeast + “Pingo” (1.3 and 2.08 mg GAE/g) and yeast mixed inoculum cheeses (1.2 mg and 2.03 mg of GAE/g). However, yeast mixed inoculum cheeses had a phenolic compound concentration of 2.87 mg GAE/g, which was significantly greater than that of the other cheeses at 45 days of maturation (Figure 2A).

Phenolic compounds are generally produced through secondary metabolism of plants and are found in low concentrations in cheeses due to animal feed. However, it has already been reported that some bacteria or even yeast can produce phenolic compounds such as xanthones, which may explain the increase in concentrations observed during maturation [25]. Phenolic compounds are responsible for changes in flavor that, depending on the concentration, can become unpleasant [26]. However, many phenolic compounds have important biological activities, and in some cases, the concentration of phenolic compounds can positively correlate with antioxidant activity, as was observed for cheese containing mixed yeast culture at 45 days of maturation [13].

The antioxidant activity, evaluated by the ABTS method, is shown in Figure 2B. The yeast mixed inoculum cheese showed greater free radical scavenging activity (49.54, 72.62 and 77.53%) at 15, 30 and 45 days of maturation, respectively.

Some authors have reported that antioxidant activity is affected by the concentrations of macro- and micronutrients, enzyme systems, hydrolyzed proteins and free amino acids. Furthermore, some microorganisms can release bioactive peptides through the metabolism of their precursor proteins through the proteolytic system during cheese maturation [27]. The results indicate that the presence of mixed yeast culture in the cheese may have had a positive impact on this aspect. Abadía-García et al. [28] and Mushtaq et al. [29] suggested that the addition of starter probiotic bacterial strains to cottage cheese increased the levels of antioxidant activity since these strains were able to metabolize polypeptides in cheese, generating peptides or free amino acids with antioxidant characteristics.

In addition, the use of starter cultures increases metabolic activity in cheese so that yeasts have greater antioxidant activity than bacteria [30,31], and this activity may be related to cell wall polysaccharides, intracellular enzymes and bioactive peptides. It has already been described that (1→3) β-d-glucan and others found in the cell walls of some yeasts may play a role in this process [32].

The general results suggest that, due to the increase in total phenolic compounds and antioxidant activity observed by the ABTS test, cheeses produced with mixed cultures of potentially probiotic yeasts have greater antioxidant capacity after 30 days of maturation. Furthermore, the need for a longer maturation period for artisanal cheeses produced with raw milk associated with the benefits related to the inclusion of probiotic yeasts demonstrates that our cheeses are in accordance with the basic needs for the commercialization of Canastra cheese.

### 3.4. Scanning Electron Microscopy

**Figure 3:**
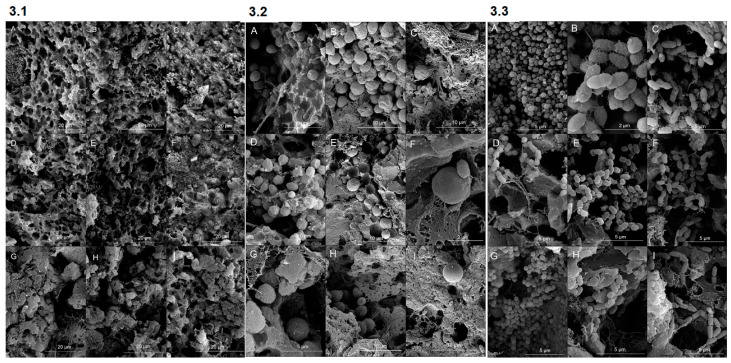
Electron microscopy of cheeses containing mixed yeast inoculum, “Pingo” and mixed inoculum and “Pingo” at 15, 30 and 45 days of maturation. 3.1 (**A**–**I**): Microstructure of the three cheeses (horizontal) throughout the maturation time (vertical). 3.2 (**A**–**I**): Yeasts present in the dough of the three cheeses (horizontal) throughout the maturation time (vertical). 3.3 (**A**–**I**): Bacteria present in the dough of the three cheeses (horizontal) throughout the maturation time (vertical).

The microstructure of the cheeses was analyzed after 15, 30 and 45 days of maturation to evaluate the effect of adding the mixed yeast culture on the structural arrangement of the cheese matrix. Electron micrographs show different microstructural arrangements depending on the maturation time and the inoculum used (Figure 3).

At 15 days of maturation, the yeasts mixed inoculum and yeasts + “Pingo” cheeses showed uniform and noticeable pores (Figure 3(3.1,3.2)). These results are probably related to the intense metabolic activity in cheeses inoculated with the mixed yeast culture. The faster acidification resulting from lactose metabolism (Table 1) by yeasts probably resulted in a faster decrease in pH, with consequent dissolution of the casein minerals. This factor likely resulted in reduced interactions between proteins and smaller casein aggregates. A greater tendency for various spaces between the protein matrix was observed [33]. On the other hand, the “Pingo” cheese had a more compact microstructure than did the other cheeses after 15 days of maturation (Figure 3(3.1)). However, at 30 and 45 days, the structure appeared less compacted and had more space (Figure 3(3.1 F and I)). This occurs because the cheese matrix with higher concentrations of lactic acid generates clots with smaller and more demineralized casein particles, resulting in a more fragile protein network [34]. These results are in agreement with the data presented in Table 1, in which lactic acid concentrations in “Pingo” cheeses were greater at 30 and 45 days than those in yeasts mixed inoculum cheeses. Furthermore, it has already been reported by Oluk [33] that some of the empty spaces sometimes represent spaces originally occupied by fat globules which were expelled during pressing.

At 45 days of maturation, a reduction in uniform distribution and an increase in pore diameter were observed in cheeses produced with yeast. As the maturation period increases, proteolytic activity decreases and can result in fewer spaces and protein breakdown, resulting in a more compact network [33,35]. This can also be explained by the decrease in lactic acid concentration as the maturation period increases in the yeast mixed inoculum and yeast + “Pingo” cheeses (Table 1). Furthermore, it is possible to observe the distribution of yeast cells throughout the cheese matrix. The yeast density was maintained during the 45 days of maturation in the yeast mixed inoculum and yeast + “Pingo” cheeses (Figure 3(3.2 G and H)). Yeast cells were generally observed in clusters. Furthermore, it can be seen through the scars that many cells were in the recent process of division, indicating that even at the end of the maturation period, these microorganisms showed intense metabolic activity, which is in agreement with the data presented in Figure 1A.

**Figure 4:**
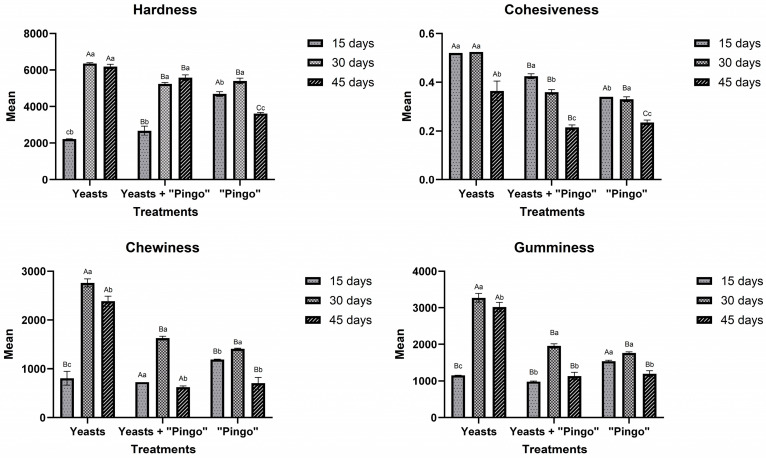
Evaluation of hardness, chewiness, cohesiveness and gumminess of cheeses containing the mixed yeast inoculum, “Pingo” and mixed inoculum and “Pingo” at 15, 30 and 45 days of maturation. Data are expressed as the mean of triplicates ± standard deviation. Means with different letters differ significantly according to Tukey’s test, *p* < 0.05. Distribution of uppercase letters of treatments over time. Lowercase letters indicate times within the treatment.

The yeast population in “Pingo” cheeses was not observed at a high density (Figure 3(3.2 I)), which may have occurred because in some situations, such as competition for nutrients, yeasts tend to migrate to specific regions of the cheese, such as the rind, which can make observation difficult depending on the sample used [36]. Bacteria in the form of cocci and bacilli could be observed, as well as the division ring, which was very present even at 45 days of maturation, demonstrating intense cell division activity (Figure 3(3.3 G and I)). It is possible to observe bacilli with fimbriae, a notable characteristic of contaminating bacteria in cheese processing [37]. Some studies have reported that the inclusion of probiotic cultures in cheeses can affect their textural characteristics, such as hardness [17]. Microscopy revealed that, in fact, the mixed yeast culture had a positive impact on this aspect since the cheeses that presented the mixed yeast culture as inoculum had a less compact matrix, one of the aspects that may have been responsible for the lower hardness values observed in these cheeses after 15 days of maturation (Figure 4).

### 3.5. Texture Analysis

The texture profiles of the cheeses after 45 days of maturation are presented in Figure 4. At 15 days, the yeast mixed inoculum cheese (2220 N) and the yeast + “Pingo” cheese (2683 N) presented lower hardness than the “Pingo” cheese (4688 N). At 30 days of maturation, an inversion in these values was observed, where the hardness values of the yeast + “Pingo” and “Pingo” cheeses were lower (5245 and 5404 N, respectively) than that of the yeast mixed inoculum cheese (6348 N) (Figure 4). At 45 days, the texture of the yeasts mixed inoculum cheese did not significantly differ from that at 30 days of maturation. Interestingly, “Pingo” cheese had the lowest hardness (3618 N). Although some research shows that the presence of probiotic cultures can improve textural aspects such as hardness, other factors can also interfere [23,38]. Kondyli et al. [39], for example, reported that a relatively low pH, resulting from factors such as the metabolism of starter cultures, may be responsible for a cheese that is less firm, more brittle and has a consequent decrease in hardness. These results, associated with higher concentrations of lactic acid at 30 and 45 days in “Pingo” cheeses, may explain the lower hardness values in the respective treatments at the end of maturation (Table 1). In general, the hardness values remained approximately stable between 30 and 45 days of maturation. Furthermore, as shown in Figure 3, the cheeses that contained the mixed yeast culture presented more noticeable macroscopic pores, probably resulting from, among other factors, the CO_2_ produced by the microorganisms. This may have influenced the reduction in the hardness of these cheeses [17].

Cohesiveness, which measures the strength of the internal bonds of the protein matrix, was also analyzed. In the three cheeses produced, the cohesiveness decreased with increasing maturation (Figure 4). Most likely, the proteolysis that occurs throughout the cheese maturation process or a possible decrease in pH caused by the increase in the activity of lactic acid bacteria may have influenced the decrease in the integrity and cohesion of the casein micelles, generating lower values with increasing maturation [40].

Chewability, characterized as the energy required to bring the cheese to a uniform state before swallowing, was positively correlated with hardness. Up to 30 days of maturation, the chewability of the yeast mixed inoculum and yeast + “Pingo” cheeses significantly increased (*p* < 0.05) over time. However, at 45 days, there was a significant decrease in chewability (*p* < 0.05), which is in line with Jia et al. [41]. “Pingo” cheese presented similar values at 15 and 30 days, with a subsequent decrease at 45 days.

All the cheeses showed an increase in gumminess up to 30 days of maturation. However, at 45 days, the gumminess decreased in all the cheeses (Figure 4). As gumminess is the product of hardness and cohesiveness, and as all cheeses showed an increase in hardness up to 30 days, it was expected that the gumminess of the cheeses would be greater at 30 days than at 45 days of maturation [39].

### 3.6. Profile of Volatile Compounds

**Figure 5:**
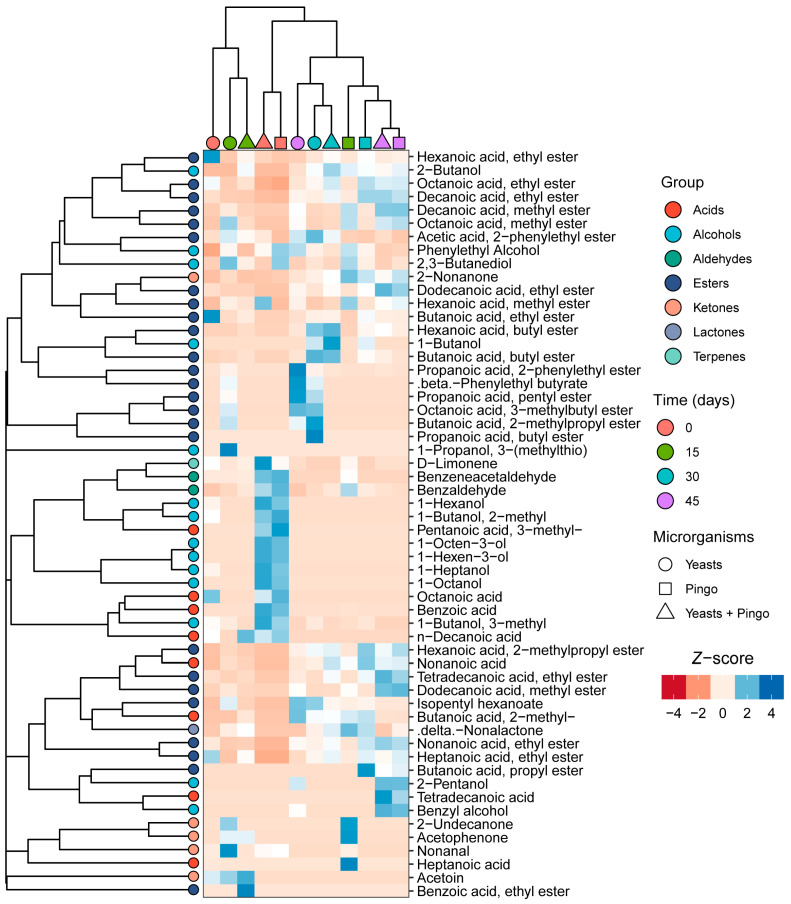
Heatmap and hierarchical cluster analysis of the volatile organic compound profile in cheeses produced with yeast mixed inoculum, mixed inoculum + “Pingo” and with only “Pingo” at 15, 30 and 45 days of maturation. The columns contain the cheeses and their respective aging times. The correlation matrix is represented by a color gradient in which dark blue indicates a positive correlation and dark red indicates a negative correlation (Pearson’s correlation coefficient). The color matrix results in the clustering tree shown in the upper part of the figure, which groups the cheeses that do not differ significantly from each other in the same branch and separates the cheeses that differ significantly in different branches.

The volatile compounds of the cheese were analyzed over the 45 days of maturation and are shown in Figure 5. Fifty-six compounds belonging to the following groups were identified: esters (25), alcohols (14), acids (8), ketones (5), aldehydes (2), terpenes (1), lactones (1). Cluster analysis showed a trend towards higher concentrations of esters at the final times of maturation (30 and 45 days), while acids, alcohols, aldehydes and ketones are predominantly observed at the initial times in all cheeses (Figure 5).

Esters were the compounds that showed the greatest abundance in all cheeses. In general, a trend toward an increase in the concentration of esters in cheeses was observed over 45 days of maturation, with an emphasis on yeasts mixed inoculum and yeasts + “Pingo” cheeses at 30 and 45 days (Figure 5). Esters are produced from the reaction between the alcohol produced by lactose fermentation or amino acid catabolism and intermediates from the catabolism of fatty acids or amino acids [42]. Some esters, such as 3-methylbutyl octanoate (-), phenethyl butyrate (fruity/floral), phenethyl propionate (roses/floral/honey), isobutyl butanoate (-), pentyl propionate (-) and butyl propionate (-), were identified in cheeses that contained the mixed yeast culture (Figure 5). Among these esters, phenethyl butyrate has previously been described in Cebreiro cheeses inoculated with the yeast *K. lactis*, which suggests that this ester is related to the metabolism of this yeast [17]. Phenethyl acetate (rose/floral), although present in all cheeses, was found to be more abundant in cheeses containing the mixed yeast inoculum at all times analyzed (Figure 5). Ethyl esters of hexanoic, butanoic, decanoic, octanoic and dodecanoic acids were the most abundant. Furthermore, they were present in all cheese samples. These esters showed fluctuations in their concentrations throughout the 45 days of maturation. The esters of hexanoic, butanoic and octanoic acids have also been described as the most abundant in goat cheese samples, contributing a sweet flavor (fruity/ice cream) and playing an important role in the aroma and flavor of several cheeses already described. However, they may present negative effects depending on the concentration [41]. Other methyl esters of decanoic, hexanoic, octanoic and dodecanoic acids were also observed in lower concentrations.

An important alcohol for the development of cheese aroma is 2-butanol, which was observed mainly in cheeses containing “Pingo” (7.07, 6.31 and 6.88%) and in yeast + “Pingo” cheeses (6.44, 9.05 and 5.24%) at 15, 30 and 45 days, respectively. In cheeses containing only the mixed culture, it was observed only after 30 and 45 days and in lower percentage (6.32 and 2.70%) (Figure 5). On the other hand, 1-butanol was observed only after 30 days of maturation in all cheeses, especially the cheese containing both inocula (3.82%) (Figure 5).

The alcohols 1-octanol and 1-octen 3-ol were observed only in cheeses containing “Pingo” and in yeasts + “Pingo” cheeses at time 0 of maturation, indicating that this alcohol is probably related to the metabolism of endogenous organisms in “Pingo”. The alcohols 1-hexanol and 1-heptanol were observed at time 0 of maturation in all cheeses; however, higher concentrations were observed yeasts + “Pingo” cheeses.

Although there are some variations in the concentrations of alcohols over the days of maturation, it is possible to observe that there is a tendency towards the reduction of these compounds over time. According to Delgado et al. [40] and Ianni et al. [42], some alcohols may present decreasing concentrations over the maturation time as many of these compounds are involved in reactions together with acids to form esters.

In cheeses, the metabolism of lactose and some amino acids, in addition to the reduction of methyl ketones and linoleic and linolenic acids, can produce alcohols of different types. Among the alcohols observed, 3-methyl-1-butanol (fresh/alcohol aroma) and phenylethyl alcohol (rose/floral aroma) were present in all cheese samples and at all maturation times (Figure 5). 3-methyl-1-butanol showed a trend of gradual decreasing concentrations over time in all cheeses and has been described as responsible for the light aroma of beef mozzarella, which provides a pleasant aroma to fresh cheese [40]. On the other hand, the phenylethyl alcohol concentration increased in the yeasts mixed inoculum cheeses, with a greater concentration observed at 45 days than at 15 and 30 days (15.94%). In the “Pingo” cheeses, this concentration of this alcohol decreased over time, and in the yeast + “Pingo” cheeses, there were variations in the concentration, where higher percentages were observed at 0 and 30 days. Centeno et al. [17] also reported significantly higher concentrations (*p* < 0.05) of this alcohol in cheeses produced with *K. lactis*. Phenylethyl alcohol is known for its pleasant floral, fruity and fermented (wine) aroma and is produced by yeast through phenylalanine [43]. This explains the greater abundance of this alcohol observed in cheeses containing mixed yeast culture.

In general, fatty acids do not make a major sensory contribution to cheeses but are related to the production of important compounds such as alcohols, esters, and methyl ketones. Octanoic acid (rancid/fatty) and benzoic acid (-) were detected in all cheeses at time 0 of maturation, with greater abundance being observed in “Pingo” (16.16%) and yeast + “Pingo” cheeses (12. 87%), respectively. Similar results were obtained by Centeno et al. [17], who observed higher concentrations of octanoic acid in cheeses containing bacterial inocula. n-Decanoic acid was also detected in all cheeses at 0 and 15 days in the yeasts + “Pingo” treatment group. These acids were detected in samples of fresh goat cheese aged 90 days and have been determined to be key compounds in the development of the flavor of this type of cheese [44,45]. 2-Methylbutanoic acid (a fruity fatty acid) was detected at all maturation times in the yeast + “Pingo” and “Pingo” cheeses. In yeast mixed inoculum cheeses, this acid was observed only at 30 and 45 days. According to previous research, butanoic acid is one of the main contributors to several cheese varieties due to its low perception threshold [46].

Aldehydes are important compounds in cheese flavor due to their low perception threshold. However, most of the time, these compounds vary greatly during cheese maturation, and according to Li et al. [47], they are quickly reduced to primary alcohols or oxidized, which may explain the absence of these compounds in some of the maturation intervals evaluated. Among the aldehydes observed, benzaldehyde (almond/cherry) was present throughout the maturation of “Pingo” and, consequently, of yeast + “Pingo” cheeses, with a decrease occurring over 45 days. In yeast mixed inoculum cheese, this compound was only present at 15 and 30 days, with lower concentrations (0.21 and 0.23%, respectively) than the concentrations observed in other cheeses. Benzeneacetaldehyde (floral/honey) was detected at 0 and 15 days in all cheeses, especially in “Pingo” cheeses (2.30 and 0.96%).

Another class of volatile compounds important for the development of cheese flavor is ketones, which are normally formed through fatty acids present during maturation. Due to their low perception threshold and characteristic aroma, ketones and, essentially, methyl ketones contribute positively to the aroma of cheeses. Fruity, floral and musty notes are associated with several methyl ketones [43]. In the cheeses evaluated, two methylketones (2-nonanone and 2-undecanone) were identified. The 2-nonanone (fruity/musty) level was greater in “Pingo” (3.68%), followed by yeast + “Pingo” (2.64%) and yeast mixed inoculum (1.62%) cheeses, after 45 days of maturation. Furthermore, an increase in their concentrations was observed over time. On the other hand, low concentrations of 2-undecanone (fruity/floral) were observed only after 15 days of ripening in the yeasts mixed inoculum and “Pingo” cheeses. Interestingly, in the yeast + “Pingo” cheeses, this compound was not detected. Compounds such as methyl ketones can cause cheese to have a sweet, waxy ester aroma, and, depending on their concentration, can have a positive impact on the aroma and flavor of the cheese [47].

Acetoin is the most abundant ketone and normally has a buttery, creamy flavor and fermented milk aroma. Acetoin was detected in only the yeast mixed inoculum and yeast + “Pingo” cheeses at concentrations of 22.62 and 32.28%, respectively, after 15 days of maturation. Similar results were found by Jia et al. [41] and Gao et al. [48], who linked acetoin to 80% of the contribution to flavor development in cheeses. This compound is directly related to 2,3-butanediol, since some yeasts use this compound as an intermediate in this process [10].

The only terpene identified was d-limonene, which, according to Ricci et al. [49], is associated with the secondary metabolism of plants, although lactic acid bacteria can also synthesize them. However, the contribution of d-limonene to the aroma and flavor of cheese is not fully defined [50].

Lactones are formed from the degradation of fat present in milk. They have creamy, sweet and fruity notes and can contribute to the flavor of cheese [51]. The only lactone observed was δ-nonalactone (coconut) at 15 and 30 days of maturation in all cheeses, especially in cheese produced with “Pingo” and in that produced with mixed yeast inoculum + “Pingo”. In general, the concentrations of this compound were relatively low.

## 4. Conclusions

The mixed culture of *T. delbrueckii* B14 and *K. lactis* B10 had a positive impact on the characteristics of the cheeses, increasing the antioxidant activity and sugar consumption associated with lactic acid metabolism. Furthermore, the inclusion of yeast influenced the emergence of aromatic compounds that positively impact the aroma and flavor of the cheese, which demonstrates that the addition of the mixed culture of *T. delbrueckii* B14 and *K. lactis* B10 did not have a negative impact on this aspect and proves to be an interesting alternative considering the probiotic potential of the yeasts used. Therefore, we conclude that the addition of the probiotic culture did not affect the quality of the cheese; however, new in vitro and, especially, in vivo studies are necessary to expand the knowledge about the influence of these yeasts on the functional characteristics of Canastra cheese.

## Figures and Tables

**Table 1:** Concentrations (g/kg) of carbohydrates and acids in cheese after 45 days of maturation.

Inoculum			Carbohydrates and Acids (g/kg)				
	Time	Lactose	Galactose	Glucose	Latic Acid *	Acetic Acid *	Citric Acid
Yeasts	0	44.5 ± 6.1 ^Aa^	Nd	Nd	3.5 ± 2.1	0.76 ± 0.1	1.6 ± 0.4 ^Ba^
	15	3.2 ± 0.9 ^Ab^	Nd	Nd	26.8 ± 6.7	1.67 ± 0.3	0.25 ± 0.02 ^b^
	30	1.5 ± 0.3 ^Ab^	Nd	Nd	1.2 ± 1.2	1.0 ± 0.1	0.04 ± 0 ^b^
	45	1.6 ± 0.01 ^Ab^	Nd	Nd	0.6 ± 0.8	1.16 ± 0.1	0.04 ± 0 ^b^
Yeasts + “Pingo”	0	44.4 ± 3.4 ^Aa^	Nd	Nd	16.7 ± 0.02	0.63 ± 0.01	1.80 ± 0.05 ^Ba^
	15	3.2 ± 0.4 ^Ab^	Nd	Nd	24.7 ± 4.2	1.53 ± 0.1	0.04 ± 0 ^b^
	30	3.9 ± 1.1 ^Ab^	Nd	Nd	14.3 ± 19.7	1.28 ± 0.2	0.04 ± 0 ^b^
	45	4.2 ± 1.3 ^Ab^	Nd	Nd	20.8 ± 2.2	1.71 ± 0.2	0.54 ± 0.7 ^b^
“Pingo”	0	44.7 ± 9.3 ^Aa^	Nd	Nd	28.7 ± 6.0	0.79 ± 0.1	2.6 ± 0.4 ^Aa^
	15	3.9 ± 0.1 ^Ab^	Nd	Nd	36.6 ± 0.4	1.86 ± 0.1	0.04 ± 0 ^b^
	30	5.6 ± 0.4 ^Ab^	Nd	Nd	41.1 ± 3.5	1.16 ± 0	0.42 ± 0.1 ^b^
	45	5.2 ± 0.04 ^Ab^	Nd	Nd	40.5 ± 4.1	1.81 ± 0.3	0.04 ± 0 ^b^

Means with different letters are significantly different according to the Tukey test, *p* < 0.05. Capital letters represent the breakdown of treatments within time, and lowercase letters represent the breakdown of times within the treatment. * Interaction not significant. Nd: Not detected.

## Data Availability

The original contributions presented in this study are included in the article. Further inquiries can be directed to the corresponding author.
